# Data mining: traditional spring festival associated with hypercholesterolemia

**DOI:** 10.1186/s12872-021-02328-4

**Published:** 2021-11-06

**Authors:** Danchen Wang, Yutong Zou, Honglei Li, Songlin Yu, Liangyu Xia, Xinqi Cheng, Ling Qiu, Tengda Xu

**Affiliations:** 1 Department of Laboratory Medicine, Peking Union Medical College Hospital, Chinese Academy of Medical Sciences, No. 1 Shuaifu Yuan, Dongcheng District, Beijing, 100730 China; 2 State Key Laboratory of Complex Severe and Rare Diseases, Peking Union Medical College Hospital, Chinese Academy of Medical Science and Peking Union Medical College, Beijing, 100730 China; 3 Department of Health Medicine, Peking Union Medical College Hospital, Chinese Academy of Medical Sciences, No. 1 Shuaifu Yuan, Dongcheng District, Beijing, 100730 China

**Keywords:** Lipid profile, Hypercholesterolemia, Chinese, Dyslipidemia, Spring festival

## Abstract

**Background:**

Serum lipid concentrations are affected by long-term high-fat diets; thus, we hypothesize that lipid levels increase after the Spring Festival in China.

**Method:**

In total, 20,192 individuals (male: n=10,108, female: n=10,084) were enrolled in this retrospective cross-sectional study based on clinical data from the Laboratory Information System (LIS) and Hospital Information System (HIS) in Peking Union Medical College Hospital from 2014 to 2018. Total cholesterol (TC), triglycerides (TGs), high-density lipoprotein cholesterol (HDL-C), and low-density lipoprotein cholesterol (LDL-C) were analyzed.

**Results:**

The serum TC [male vs. female: (4.71 ± 0.90 vs. 4.56 ± 0.85) mmol/L], TG [male vs. female: (1.71 ± 1.56 vs. 1.02 ± 0.68) mmol/L], and LDL-C [male vs. female: (3.01 ± 0.77 vs. 2.73 ± 0.74) mmol/L] levels were significantly higher in males than in females (P < 0.001); serum HDL-C [male vs. female: (1.18 ± 0.28 vs. 1.50 ± 0.34) mmol/L] was significantly lower in males (P < 0.001). In February, the TC, TG, and LDL-C levels were 8.4%, 16.3%, and 9.3% higher than the lowest levels recorded, respectively. The prevalence of dyslipidemia of the two weeks before the Spring festival was significantly lower than that of the first week after the Spring festival (43.6% (168/385) vs. 54.1% (126/233), P=0.007). Additionally, the prevalence of dyslipidemia was statistically higher in the first week after the Spring Festival than in May–January.

**Conclusion:**

Higher TC, TG, and LDL-C in winter could be associated with high-fat diets during the Spring Festival. The Spring Festival was immediately followed by a higher lipid concentrations. Thus, we don't recommend lipid assessment or physical examination immediately after the holiday especially Spring festival.

**Supplementary Information:**

The online version contains supplementary material available at 10.1186/s12872-021-02328-4.

## Background

Lipid profiles, including TC, TG, HDL-C, and LCL-C, are primary analytes for the diagnosis of dyslipidemia in clinical settings. A seasonal variation in lipid profiles has been reported by several studies in various countries [1–7], with higher serum lipid levels during the winter months than in the summer [1–4, 7]. A longitudinal study enrolled 517 healthy volunteers with a baseline TC of 5.75 mmol/L in men and 5.52 mmol/L in women [8] and found that the amplitude of seasonal variation in TC was 0.10 mmol/L (peaked in December) in men and 0.14 mmol/L (peaked in January) in women [8]. Moreover, a cross-sectional study enrolled 245 healthy young students (110 men and 135 women) using a consignor analysis and found that the TC, TG, HDL-C, and LDL-C levels exhibited statistically significant seasonal patterns [9].

Similarly, our previous study reported seasonal variation in lipid profiles, with peak values during the winter and decreasing levels in summer [4]. Although the mechanism for this seasonal variation is unclear, we suspect that in China, specifically, the increased lipid levels are associated with high-fat diets during the Spring Festival, a traditional festival in China. During this time, family members gather to celebrate, often eating high-fat meals, including pork, beef, lamb, and chicken, which may contribute to increased serum lipid levels. A meta-analysis revealed that long-term high-fat diets are associated with increased blood lipid levels [10]. Fatty diets also have a significant influence on blood lipid concentrations in overweight or obese individuals [11]. Alternatively, carbohydrate-restricted diets have been reported to be associated with decreased LCL-C in overweight and obese adults [12]. Similarly, a randomized general community trial reported that replacement of saturated fats with carbohydrates from grains, vegetables, legumes, and fruits reduces TC and LCL-C levels. Furthermore, an increasing number of studies are currently focusing on the big data of laboratory medicine to explore trends for a variety of common analytes by months or years.

Accordingly, in this study, we hypothesize that the TC, TG, and LDL-C levels increase along with higher rate of dyslipidemia, while HDL-C decreases during the first week after the Spring Festival, based on clinical laboratory big data.

## Materials and methods

### Data collection

A total of 280,206 records were derived from the Laboratory Information System (LIS) and Hospital Information System (HIS) of Peking Union Medical College Hospital from 2014 to 2018. The inclusion and exclusion criterion schematic is described in our previous study [4]. Ultimately, a total of 20,192 individuals were enrolled for further analyses.

### Laboratory measurements

Serum total cholesterol (TC), triglyceride (TG), high-density lipoprotein cholesterol (HDL-C), low-density lipoprotein cholesterol (LDL-C), total protein (TP), albumin (Alb), total bilirubin (TBil), direct bilirubin (DBil), alanine aminotransferase (ALT), aspartate aminotransferase (AST), creatinine (Cr), uric acid (UA), and glucose (Glu) were measured with a Roche C8000 automatic analyzer (Roche, Basel, Switzerland).

### Definition of the spring festival and dyslipidemia

According to the traditional festival in China, the first day of the lunar calendar marks the beginning of the Spring Festival. In China, seven days are allocated to the celebration of this festival from the last day of December to January 6^th^ according to the lunar calendar. Thus, the timing of the Spring Festival differs from year to year. The dates of the Spring Festival during the study period are shown in Table 1.

**Table 1:** Date of spring festival from 2014 to 2018

Year	Spring festival
2014	31, Jan–6, Feb
2015	18, Feb–24, Feb
2016	7, Feb–13, Feb
2017	27, Jan–2, Feb
2018	15, Feb–21, Feb

The four seasons were defined as follows: spring (March, April, and May), summer (June, July, and August), autumn (September, October, and November), and winter (December, January, and February).

Dyslipidemia was defined as TC ≥ 5.2 mmol/L, TGs ≥ 1.7 mmol/L, HDL-C < 1.0 mmol/L or LDL-C ≥ 3.4 mmol/L [13]. The non-High density lipoprotein cholesterol (non-HDL-C) concentration  = TC concentration - HDL-C concentration.

### Statistical analysis

Data were analyzed using SPSS 20.0 software (SPSS Inc., Chicago, IL, USA), Excel 2010 (Microsoft Inc., USA) and GraphPad Prism for Windows (GraphPad Software, San Diego, CA). A Kolomogorov-Smirnov analysis was used to evaluate the distribution of data. Normally distributed data are presented as the mean ± standard deviation, while nonnormally distributed data are expressed as the median and quartiles. Kruskal–Wallis or Mann–Whitney U tests were used to compare the differences. A chi-squared test was used to analyze the differences in the prevalence of dyslipidemia by month. P values < 0.05 were considered statistically significant.

## Results

### Basic characteristics of enrolled population

**Fig. 1:**
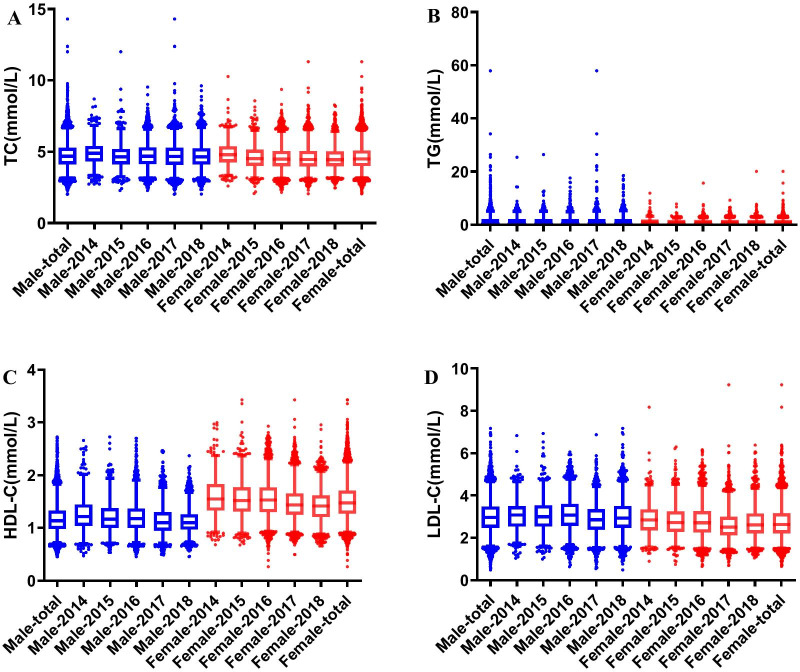
Basic concentrations of lipids of the enrolled population by sex. **A**–**D** represent the TC, TG, HDL-C, and LDL-C distribution by sex. The x axis represents the subgroup by sex (blue represents male, red represents female) and years; the y axis represents the average concentrations of lipid profiles

In total, 20,192 individuals, including 10,108 males and 10,084 females, were enrolled in this study. The average age, BMI, SBP, and DBP were 39.4 years, 23.7 kg/m^2^, 118 mmHg, and 73 mmHg, respectively. The basic characteristics of the enrolled participants by season are shown in Table 2. Differences were observed in the serum TP, Alb, TBil, DBil, ALT, ALP, Glu, UA, and Cr levels by seasons (all P > 0.05). The lipid concentrations by sex are shown in Fig. 1. The serum TC [male vs. female: (4.71 ± 0.9 vs. 4.56 ± 0.85) mmol/L], TG [male vs. female: (1.71 ± 1.56 vs. 1.02 ± 0.68) mmol/L], and LDL [male vs. female: (3.01 ± 0.77 vs. 2.73 ± 0.74) mmol/L] levels were significantly higher in males than in females (P < 0.001); serum HDL-C [male vs. female: (1.18 ± 0.28 vs. 1.50 ± 0.34) mmol/L)] was statistically lower in males (P < 0.001).

**Table 2:** Basic characteristics of the enrolled population by season

Analytes	Spring (n = 4819)			Summer (n = 6418)			Autumn (n = 5530)			Winter (n = 3425)			Total (n = 20,192)			P value
	Median	*P*_*25*_	*P*_*75*_	Median	*P*_*25*_	*P*_*75*_	Median	*P*_*25*_	*P*_*75*_	Median	*P*_*25*_	*P*_*75*_	Median	*P*_*25*_	*P*_*75*_	
Age (years)	40	31	49	37	30	47	36	29	46	37	30	46	37	30	47	< 0.001
BMI (kg/m^2^)	23.8	21.3	26.3	23.2	21.0	25.9	23.0	20.6	25.6	23.5	21.1	26.3	23.4	21.0	26.0	< 0.001
SBP (mmHg)	116	106	128	115	105	128	114	104	127	117	106	130	115	105	128	< 0.001
DBP (mmHg)	73	66	80	72	66	79	72	66	79	74	67	81	73	66	80	< 0.001
TP (g/L)	72	70	75	72	70	75	73	71	76	73	70	75	73	70	75	< 0.001
Alb (g/L)	62	60	64	62	60	64	62	60	64	62	60	64	62	60	64	< 0.001
Tbil (μmol/L)	10.5	8.1	13.9	10.9	8.2	14.7	10.6	8.1	13.9	10.3	7.8	13.5	10.6	8.1	14.1	< 0.001
Dbil (μmol/L)	4.1	3.3	5.1	4.1	3.3	5.3	4.0	3.2	5.1	3.9	3.2	4.9	4.1	3.3	5.1	< 0.001
ALT (U/L)	17	12	26	17	12	25	17	12	26	18	13	27	17	12	26	< 0.001
ALP (U/L)	60	50	72	60	50	71	59	49	71	60	50	71	60	50	72	0.009
AST (U/L)	18	15	22	18	16	22	18	15	22	18	15	22	18	15	22	0.231
Glu (mmol/L)	5.0	4.7	5.4	5.0	4.7	5.3	4.9	4.7	5.3	5.0	4.7	5.4	5.0	4.7	5.3	< 0.001
UA (μmol/L)	307	250	373	318	260	384	299	246	367	297	244	364	306	251	374	< 0.001
Cr (μmol/L)	72	61	84	72	61	84	67	58	80	71	60	82	70	60	82	< 0.001

Data are expressed as median and quartiles. The P value presents the differences by seasons. BMI: body mass index; SBP: systolic blood pressure; DBP: diastolic blood pressure; TP, total protein; Alb, albumin; TBil, total bilirubin; DBil, direct bilirubin; ALT, alanine aminotransferase; ALP, alkaline phosphatase; AST, aspartate aminotransferase; Glu: glucose; UA: uric acid; Cr, creatinine

### Distribution of lipid profiles by months

**Fig. 2:**
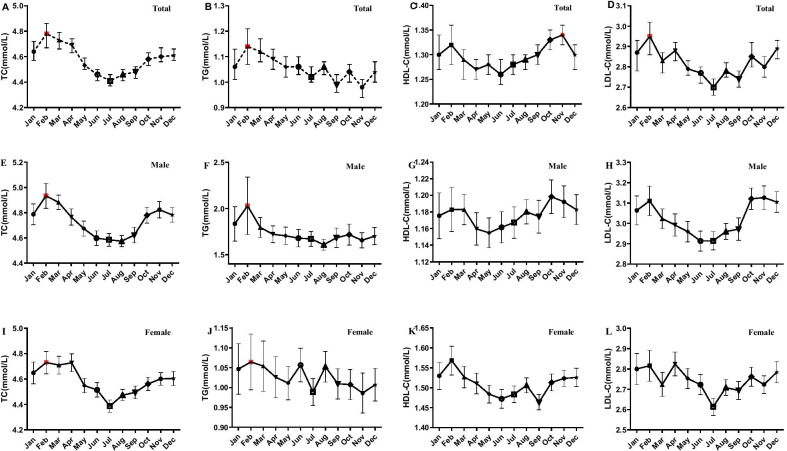
Distribution of lipid profiles by month and sex. A to D represent the distributions of TC, TG, HDL-C and LDL-C by month in total; E to H represent the distributions of TC, TG, HDL-C, LDL-C by month in male; I to L represent the distributions of TC, TG, HDL-C, LDL-C by month in female

The serum TC, TG, HDL-C, and LDL-C distributions by month and sex are shown in Fig. 2. The highest levels of TC, TG, and LDL-C were observed in February, while HDL-C was highest in November. The highest levels of TC and TG were also observed in February in males and females. Importantly, the first week after the Spring Festival occurred in February. The deviations between the lowest and highest TC, TG, HDL-C, and LDL-C were 8.4%, 16.3%, 6.3%, and 9.3%, respectively.

### Distribution of lipid profiles between the first week after the spring festival and other days

**Fig. 3:**
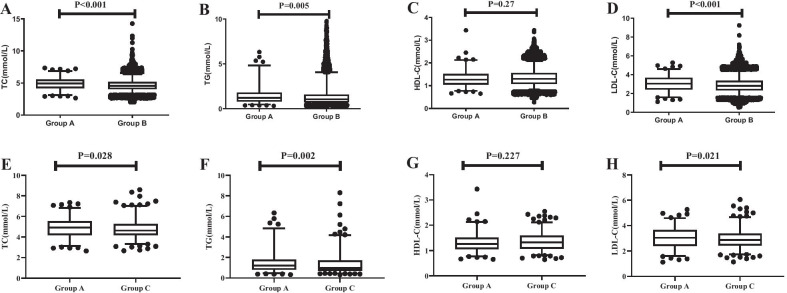
Distribution of lipid concentration by festival. Group **A** represents the first week after the Spring Festival. Group **B** represents other times of the year. Group **C** represents the two weeks before the Spring Festival. **A** to **D** represent the distribution of TC, TG, HDL-C, and LDL-C concentrations between the first week after Spring Festival and the other times of year. **E** to **H** represent the distribution of TC, TG, HDL-C, and LDL-C cocnentrations between the first week after Spring Festival and the two weeks before the Spring Festival

The distribution of lipid profiles between the first week after the Spring Festival and other days in the year is shown in Fig. 3(A to D). The serum TC, TG and LDL-C concentrations in the first week after the Spring Festival were significantly higher than those on other days (all P < 0.05). The distribution of lipid profiles between the first week after the Spring Festival and the two weeks before the Spring festival is shown in Fig. 3 (E to H). The serum TC, TG, and LDL-C concentrations in the first week after the Spring Festival were significantly higher than those in the two weeks before the Spring Festival (all P < 0.05).4 The non-HDL-C in the first week after Spring Festival was higher than that in the two weeks before Spring Festival [(3.57 ± 0.98) mmol/L vs. (3.41 ± 0.95) mmol/L, P=0.012].

### Prevalence of dyslipidemia

**Fig. 4:**
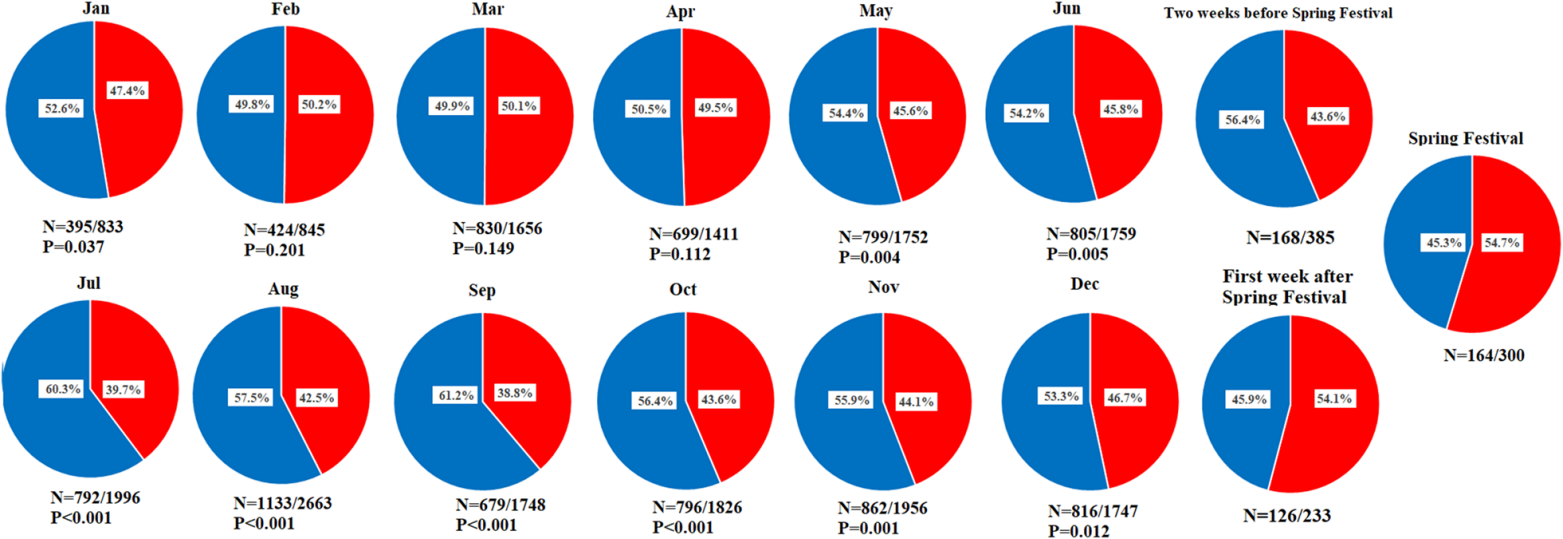
Prevalence of dyslipidemia. The red area represents the prevalence of dyslipidemia, whereas the blue area represents the prevalence of normal lipid concentrations

The overall prevalence of dyslipidemia was 44.7%, and the prevalence was significantly higher in males than in females (59.4% vs. 30.0%, P < 0.001). Moreover, the prevalence of dyslipidemia in spring, summer, autumn, and winter was 48.3%, 42.5%, 42.3% and 47.7%, respectively. After stratifying by sex, the prevalence of dyslipidemia in spring, summer, autumn, and winter was 61.5% and 32.8%, 56.3% and 28.1%, 59.7% and 28.7%, and 61.6% and 32.2% for males and females, respectively. The prevalence of dyslipidemia by month is shown in Fig. 4, and the prevalence was higher in the Spring Festival than in the other months. Compared with the Spring Festival, the prevalence in January (P = 0.037), May (P = 0.004), June (P = 0.005), July (P < 0.001), August (P < 0.001), September (P < 0.001), October (P < 0.001), November (P = 0.001), and December (P = 0.012) was statistically lower. Compared with the first week after the Spring Festival, the prevalence of dyslipidemia in January (P=0.042), May (P=0.009), Jun (P=0.01), July (P<0.001), August (P<0.001), September (P<0.001), October (P=0.002), November (P=0.002), and December (P=0.02) was significantly lower. After stratifying by sex, the prevalence of dyslipidemia between months and the first week after the Spring Festival is shown in Additional file 1: Fig. 1. The prevalence of dyslipidemia of the two weeks before the Spring festival and the first week after the Spring festival were 43.6% (168/385) and 54.1% (126/233), respectively. Otherwise, the prevalence of dyslipidemia of the first week after the Spring festival was significantly higher than that of the two weeks before the Spring festival (P=0.007).

## Discussion

Seasonal variations in lipid levels have been confirmed by many studies [1–4, 9], some of which have indicated an increased risk of cardiovascular diseases in winter due to higher lipid levels [1, 4]. In this study, we found that serum lipid concentrations were higher (lower in HDL-C) in males than in females, which may be associated with different lifestyles and eating habits. Thus, to verify the interaction of sex and seasonal variation in lipid levels, we conducted stratified analysis for seasonal variation in lipid levels based on males and females. We found that the seasonal variation in lipids appeared to differ between males and females, suggesting that the differences in lifestyles and dietary intake affect lipid metabolism [14].

Increased LDL-C levels have been described to be associated with cardiovascular disease. In fact, reduced LDL-C levels are often prescribed as an effective method to prevent cardiovascular diseases [15]. Furthermore, increased TG concentration may also be associated with cardiovascular diseases [16]. Thus, lipid therapy and management could help to prevent cardiovascular diseases [17]. National Health and Nutrition Examination Survey data from 2003 to 2006 in the United States reported that 53% of adults had lipid abnormalities, 21% of whom had dyslipidemia [15], which is an important risk factor for cardiovascular diseases [18, 19]. The prevalence of dyslipidemia was 34% (35.1% in urban and 26.3% in rural areas) based on a multistage, stratified sampling method in China [20]. China National Stroke Screening and Prevention Projects reported a similar prevalence of dyslipidemia between rural and urban populations (43.2% vs. 43.3%), which agreed with our study [21]. After stratifying by sex, we also found that the prevalence of dyslipidemia in the first week after the Spring Festival was higher than that in other months. Thus, we don't recommed assess the lipid concentrations after holiday especially Spring Festival.

In this study, we confirmed the association between increased lipid levels and high-fat diet intake during the Spring Festival, which was consistent with a Denmark study enrolling a total of 25,764 participants from the general population [1]. This observational study reported 15% higher TC and 20% higher LDL-C levels in the winter than in the summer, which was associated with high-fat diets during Christmas holidays [1]. The same trend was also observed in a French study, with 6.4% higher TC and 8.7% higher LDL-C in winter months than in summer [2]. Furthermore, the deviation in the current study between lowest and highest TC and LDL-C were 8.4% and 9.3%, respectively, which was similar to the results of a French study [2]. Interestingly, the differences of TC and LDL-C  between the first week after the Spring Festival and the other times of the year were more significant than that between the first week after the Spring Festival and the two weeks before the Spring Festival, which could be explained by the significant seasonal fluctuation of TC and LDL-C. Otherwise, the differences of TG between the first week after the Spring Festival and the two weeks before the Spring Festival were more signficant, implying that the TG are more likely affected by lifestyles especially diet. Specifically, we found the prevalence of dyslipidemia to be significantly higher in the first week after the Spring Festival than in the other months in both males and females, which was consistent with a previous study [1]. Furthermore, a cross-sectional study that analyzed data from normal-lipidemic individuals and dyslipidemic individuals found strong positive cross-correlations between TC, TG, LDL-C, and HDL-C levels, suggesting similar behavior in normal and dyslipidemic populations [22].

There are several strengths to emphasize in this study. Firstly, we used clinical laboratory big data downloaded from LIS and HIS to conduct this study, which is not only cost-effective but also efficient. Secondly, we only included information from the initial reports generated for individuals who visited the Department of Health Medicine to ensure the consistency of all data. Additionally, to avoid having sex as a confounding variable when examining the effect of Spring Festival and prevalence of dyslipidemia, we performed individual analyses for variation and prevalence by sex. Last but not least, this study was the first to report an association between increased lipid levels and the Spring Festival in China.

However, certain limitations were also noted in this study. Although we used clinical data to analyze the association between increased lipid levels and the Spring Festival, we did not collect dietary intake information. However, it is well known that during the Spring Festival, a large proportion of the population gathers to celebrate the holiday with more high-fat meals and reduced levels of exercise [23]. Moreover, information on whether participants were taking lipid-lowering therapy was unknown. Thus, to verify the association between high-fat diet intake during the Spring Festival and lipid levels, additional randomized studies are required. Otherwise, further cohort study is needed to confirm that the changes were caused by festivals. In future studies, we can recruit other groups who were not participating in the Spring Festival nationwide or potentially worldwide to confirm the association between changes in lipids and the Spring Festival.

## Conclusion

In conclusion, we confirmed that higher lipid levels are associated with a high-fat diet during the Spring Festival, with notable differences observed between males and females. However, the prevalence of dyslipidemia during the first week after spring was higher than in any other month for both males and females. Thus, to accurately diagnose dyslipidemia immediately after the Spring Festival, it is necessary to consider the effect of high-fat diet during Spring Festival. We don't recommend lipid assessment or physical examination immediately after holiday especially Spring Festival.

## Supplementary Information

**Additional file 1. Supplemental Figure 1.** Prevalence of dyslipidemia by sex and monthGroup A represent the first week after Spring Festival.

## Data Availability

The datasets generated and analyzed during the current study are available from the corresponding author Ling Qiu on reasonable request.

## Abbreviations

Alb: Albumin
ALT: Alanine aminotransferase
AST: Aspartate aminotransferase
Cr: Creatinine
DBil: Direct bilirubin
HDL-C: High-density lipoprotein cholesterol
HIS: Hospital Information System
LDL-C: Low-density lipoprotein cholesterol
LIS: Laboratory Information System
TBil: Total bilirubin
TC: Total cholesterol
TG: Triglyceride
TP: Total protein
UA: Uric acid
