# Supplementary material for: Data mining: traditional spring festival associated with hypercholesterolemia
Source: BMC Cardiovasc Disord. 2021 Nov 6;21:526. doi: 10.1186/s12872-021-02328-4 (PMC8571822; doi:10.1186/s12872-021-02328-4)
Supplement: Supplementary file 1 — Additional file 1. Supplemental Figure 1. Prevalence of dyslipidemia by sex and monthGroup A represent the first week after Spring Festival. [file 12872_2021_2328_MOESM1_ESM.docx]

**Supplemental Material**

**Supplemental Figure 1** Prevalence of dyslipidemia by sex and month

Group A represent the first week after Spring Festival
